# National or local infodemic? The demand for news in Italy during COVID-19

**DOI:** 10.1007/s10754-023-09350-3

**Published:** 2023-04-17

**Authors:** Stefano Castriota, Marco Delmastro, Mirco Tonin

**Affiliations:** 1 Department of Political Sciences, University of Pisa, Via Serafini 3, 56126 Pisa, Italy; 2 Autorità Per Le Garanzie Nelle Comunicazioni, Via Isonzo 21/B, 00198 Rome, Italy; 3 Faculty of Economics and Management, Free University of Bozen-Bolzano, Piazza Università, 1, 39100 Bolzano, Italy; 4 FBK-IRVAPP, Trento, Italy

**Keywords:** News, Local news, TV, COVID-19, D12, I19, L82

## Abstract

Information can have an important impact on health behavior and, according to the World Health Organization, an ‘infodemic’ has accompanied the current pandemic. Observing TV news viewership in Italy during the COVID-19 pandemic using actual consumption data, we investigate whether demand for national and local news depends on national or local epidemiological developments, as measured by the number of new positives or the number of current positives on any given day. Exploiting the fact that the impact of the pandemic displays a great deal of variation among the different regions, we find that at the regional level, demand for both national and local news responds to national epidemiological developments rather than to local ones. This has implications regarding the incentives for local politicians to take preventive action.

## Introduction

The coronavirus crisis has also been defined as a communication crisis (Gollust et al., 2020), and the role of information is considered central in supporting pandemic response (Van Bavel et al., 2020). In the new and fast-changing environment of the pandemic, communication plays a crucial role, as the population is asked to alter its day-to-day behavior fundamentally in order to comply with social distancing and other measures like hand washing or mask wearing. There are already several studies, reviewed here below, which show how news media affected people’s behavior during the pandemic, with some finding evidence of an effect on the spread of the virus and on mortality. The importance of media on disease spread is such that its effect has been incorporated into epidemiological models (see, for instance, Kim et al., 2019).

Information supply about the pandemic ranges from the media reporting accurate and evidence-based information – the type of media we study in this paper – to outlets spreading conspiracy theories or sensationalized fake news, giving rise to an ‘infodemic’ (World Health Organization, 2020)^1^. Studying the public’s demand for professionally-sourced information during the pandemic is therefore essential to understanding what type of information spreads, with important implications for public health and policy.

In this paper we study the demand by Italian citizens for national and local TV news during the pandemic. We show how the share of TV viewers watching the news increases as epidemiological conditions worsen – measured by the number of new positives and the number of current positives on any given day. Demand for both national and local TV news is responsive to national conditions, but not to local ones.

We use high-quality data on television usage akin to PeopleMeters^2^. Italy was the first country with a free press to be severely affected by the virus. In addition, as in other markets like the US (Pew Research Center, 2018), TV is still the dominant mass medium, and prime time TV news outlets are by and large the main source of information for Italian citizens (Agcom, 2018a). We study how consumption of local and national news changed with the evolution of the pandemic and, in particular, we analyze whether attention for local and national news depends on local or national epidemiological developments. This is particularly relevant in the Italian context where, during its first wave, COVID-19 affected the country very unevenly. For instance, Lombardy – where half of the overall deaths in the period under consideration occurred – was severely affected, with 7.5% of the population having contracted the virus as of July 2020, while Sicily was barely touched, with only 0.3% of the population infected (ISTAT, 2020). In general, the pandemic hit hard in the north, while the center and the south of the peninsula were affected only marginally.

National and local facts and policies interact differently with local and national TV news (Martin & McCrain, 2019; Yazaki, 2017). It is reasonable to assume that demand for national news would depend on topical national (and international) events, while relevant local events would drive demand for local news. For example, a national election should attract more national news viewers, while a regional or mayoral election should more strongly affect the local news viewership. Turcotte et al. (2017), for instance, show that local television news coverage of the Deepwater Horizon oil spill depends on geographical proximity, while Branton and Dunaway (2009) show how news organizations closer to the U.S.-Mexico border generate a higher volume of articles about immigration^3^. In the specific case of public health issues, it is noted that “[b]ecause many public health issues (e.g., an infectious disease outbreak, a water supply toxin, or access to grocery stores) are local in reach, local news has an opportunity to speak to community health concerns more directly than can national outlets” (Gollust et al., 2019)^4^. In the case of COVID, the pandemic quickly became a global event. Nevertheless, the first wave impacted the various regions of Italy in a very uneven way, as we will detail in the sections below. Therefore, local epidemiological developments did not at all mirror the national ones, making it possible to disentangle the impact of the two.

Thus, it is reasonable to hypothesize that viewership of national news will depend on epidemiological developments at the national level, while viewership of local news will be more strongly connected to local conditions^5^. Local news, for instance, is particularly relevant in evaluating the risks associated with going to the grocery store. National news is instead more relevant for understanding international and nation-wide developments, such as those related to government policies meant to contain the virus, e.g., when a lockdown is going to be lifted, when non-essential economic activities can resume, and so on^6^. Indeed, according to a PEW Survey conducted in April 2020^7^, American adults followed national news to obtain information on topics like the economic (46%) and health (40%) impact of COVID-19, or to seek advice from national health organizations (40%). They instead followed local news to find information about local government actions (42%), goods in local stores (38%), and the status of nearby schools (30%).

What we find in this study is that both national and local TV news viewership is not responsive to local conditions, but to conditions outside of the region^8^. Considering the previous discussion, the finding that demand for local news does not respond to local epidemiological developments can be considered quite surprising. In the conclusions, we discuss possible reasons for this result and its implications, for instance concerning the incentives for local politicians to take preventive action. Besley and Dray (2020) study the role of free media in explaining the government response to the pandemic. They show how countries with free media are more responsive to epidemiological developments: they are more likely to impose a lockdown as the death toll from COVID-19 increases, and they see greater reductions in mobility during a lockdown in response to rising deaths rates. They explain these results with the fact that citizens of free-media countries are better informed, which affects compliance and decisions to lock down. We show how demand for both local and national information is responsive to the seriousness of the pandemic at the national level, and at the end of the paper we discuss the implications for the political economy of the response.

There is ample literature, as reviewed by Wakefield et al. (2010), underlining the role of information on health behaviors (see, for instance, Brown & Schrader, 1990; Chern et al., 1995; Slade, 2012). This paper contributes to the literature on information and COVID-19. Several studies in the US have investigated the effect of the Fox News media outlet on public behavior. Ash et al. (2022) utilize random variation in Fox News viewership to show how higher viewership means that people are less likely to stay at home and consume goods like cleaning products, hand sanitizers, and masks. Simonov et al. (2021) also show the negative effect of Fox News on social distancing, while Bursztyn et al. (2022) study how exposure to Fox News shows, with their divergent coverage of the coronavirus, affect behavior and downstream health outcomes. In Italy, Deiana et al. (2022) show the impact on vaccine hesitancy of news media attention to the Vaxzevria vaccine side effects, emphasizing how the public's interest in adverse vaccine events negatively correlates with COVID cases and deaths across regions.^9^ In the context of Brazil, Ajzenman et al. (2020) use news coverage and social media data to show the negative effect of speeches by Bolsonaro on social distancing. Watanabe and Yabu (2021) show that three-quarters of the decrease in outings in Tokyo are the result of “information updating on the part of citizens through government announcements and the daily release of the number of infections” and only one quarter is due to legally binding measures. Kim et al. (2020) utilize variation for rural counties in the US to reveal that local news is focused on urban communities and to demonstrate that the impact of COVID-19 can be quite different between rural and urban communities. They show that rural residents are more likely to practice social distancing if their media market is more impacted by the pandemic due to nearby urban communities, thus underlining the importance of local information.

These studies show the impact of news on preventive behavior during the pandemic, and thus underscore the importance of studying demand for news. However, they do not study how this demand changed during the course of the COVID-19 pandemic. Also, unlike the studies that focus on biased information, we instead examine the effect of official statistics about the pandemic on the demand for news.

More generally, our study also contributes to the literature on consumer demand for media, as reviewed in Berry and Waldfogel (2015). Chiou and Tucker (2013), for instance, study the impact of paywalls on the demand for online news, while George and Peukert (2019) show that demand for both local and national news among minorities depends on the minority population at the local community level. What occurs in our context is a combined change in the outside option and a shift in tastes. The change in the outside option is due to restrictions on activities outside the home, as imposed by the lockdown, and should affect the demand for media in general; in other words, more people watch TV during the lockdown because they cannot go to the gym or to the pub. This change clearly affects the absolute number, but less so the share of TV viewers watching news. In the first wave of the pandemic, these restrictions applied to all regions as they were imposed nationwide. The shift in tastes occurs if, for instance, people derive higher utility during the pandemic from news programs compared to entertainment programs because, for example, they want to stay informed about the latest epidemiological and policy developments. The shift could also occur in the opposite direction if, for instance, due to the anxiety induced by the grim reporting of death and contagion statistics during the pandemic, people chose to isolate themselves and reduce their exposure to news. Indeed, the PEW Research Center Survey mentioned above has found that 7 out of 10 US adults feel they need to take breaks from COVID-19 news^10^. Among news programs themselves, as already discussed, there can be a differential change in demand for local compared to national news. We document the changes in demand for both types of news and investigate their relationship with epidemiological developments at the national and local level, thus contributing to our understanding of consumer demand during a pandemic.

The remainder of the paper is organized as follows. The next section briefly describes the main stylized facts about news consumption in Italy and the broad developments of the pandemic. Sect. "News consumption and COVID-19 in Italy" presents the data sources. In Sect. "Data sources and descriptive statistics", we provide descriptive statistics and the main results. The final section offers our conclusions and discusses the implications of our findings.

## News consumption and COVID-19 in Italy

The Italian news landscape is historically dominated by the presence of major TV channels. As the Italian communication authority AGCOM indicates: “[t]elevision is confirmed as the medium with the greatest informative value, both for access frequency and for perceived importance and reliability” (Agcom, 2018a). More than 90% of the Italian population accesses news through TV (Internet 70%, radio 66% and newspapers 60%), and nearly half consider it their main source of news (Internet 26%, newspapers 17% and radio 8%). The COVID-19 pandemic has reinforced the role of TV (and online outlets) as the primary source of national and local news, while newspapers and radio lost a significant audience (Agcom, 2020b), a phenomenon that has also been observed in the US^11^.

More specifically, Italians widely regard primetime (at 8 PM) national TV news as the most important time to get daily access to news and information. In particular, TG1 of RAI – the Italian public broadcaster – and TG5 of Mediaset – the main commercial broadcaster in Italy – are the two most viewed TV news outlets, reaching an average daily audience of 4 millions people each (in 2019, 4.7 millions for TG1 and 3.9 millions for TG5; Agcom 2020a). TGLa7 (TG7 from here forward) of Cairo Communication (an Italian independent news group) complements the national news offerings at primetime with TV news that reaches a much smaller audience of more educated people (Agcom, 2019)^12^.

With regards to local news, TGR, the regional TV news outlet for RAI, is by and large the main source for local news in Italy (Agcom, 2018b) and, because of this, we will use “regional news” as synonym for local news^13^. Coordinated by a central structure and maintaining a similar format, each region features a dedicated newsroom that produces and broadcasts three TV daily news programs (at 2 PM, 7:30 PM, and 00:10 AM), of which the primetime program at 7:30 PM reaches more than 2 million Italians (2.3 in 2019) and is the third TV news outlet in terms of viewership after TG1 and TG5.

Our focus on primetime TV news enables us to observe the evolution of (local and national) news viewership at a regular, fixed time, thus holding the news supply constant in terms of available programs. By comparing TV news viewership before and during the first COVID-19 pandemic wave and analyzing the relationship between news viewership and epidemiological developments, we gain a better understanding of the effects of COVID-19 on public attention to news.

Regarding COVID-19 in Italy, the Government declared a six-month state of emergency on January 31, 2020, the same day on which the first cases, among two Chinese tourists, were confirmed in Rome (see Appendix for a detailed chronology and a timeline of COVID-19 events and public policies in Italy). When splitting the sample between a pre- and post- COVID periods, we take this date as the threshold. The contagion spread more heavily and rapidly in the northern regions, with the first cases of community transmission reported in Lombardy and Veneto in late February and the imposition of a localized lockdown in the outbreak areas. Between March 8 and 9, the whole country went into lockdown. National lockdown measures were extended twice and finally ended on May 3, 2020, when a so-called “Phase 2” started nationwide, with a gradual re-opening.

An important aspect to emphasize concerning epidemiological developments in Italy is that, as we will detail in the next section, different areas have been affected in very different ways, with a clear North–South gradient. Lombardy alone, with one sixth of the Italian population, has had almost 50% of deaths and 40% of infections. Sicily, whose population is half that of Lombardy, accounted for 0.9% of deaths and 1.3% of infections (ISTAT, 2020).

## Data sources and descriptive statistics

The television viewership data used here come from Auditel, which is the organization providing quantitative data on TV viewership in Italy (similar to Barb in the UK and Nielsen in the US). It should be noted that “[o]ne defining characteristic of the audience measurement industry is that, although a number of different firms provide statistical representations of media audiences, only one firm tends to dominate the distribution of comprehensive audience data for each media technology” (Napoli, 2003). In this regard, Auditel is the only producer of audience data for the Italian TV market. It is a Joint Industry Committee (JIC), a reciprocal-control organization that brings together all TV market players, namely (national and local) broadcasters, advertisers, media agencies and media buyers. Since 1984, Auditel has performed the task of measuring and releasing data on the entire digital, satellite, live and on-demand Italian TV offerings across all platforms and devices, 24 h a day, minute by minute. Nowadays, data are provided by a panel of 16,100 households (sampled to represent the entire Italian population) distributed across all 20 Italian regions. The panel households are equipped with a meter which measures the TV viewing of the household members and any possible guests, minute by minute and every day. The meter monitors TV consumption on traditional TV, Smart TV, PC, Game console, and other devices.

We have constructed a panel with daily data for each of the 20 Italian regions, ranging from January 1, 2019 until July 27, 2020. Data are not available at a lower level of disaggregation, e.g., at the provincial or individual level. In our main analysis, for the dependent variable we use the percent share of the primetime national (8:00–8:30 PM) and local (7:30–8:00 PM) TV news, representing the share of TV viewers who are watching the news in the corresponding time slots in each region. We also have data on the absolute number of viewers, but this may be strongly affected by the fact that during lockdown, people watch more TV due to a lack of alternatives (e.g., closure of bars, limitation of interpersonal contacts). Looking at the share better isolates increased interest in the news rather than in TV more generally.

Looking at Table 1, we can see that national news (i.e., the cumulative share of the three national primetime news outlets: TG1, TG5 and TG7) in general have a high share, 47.7% on average, and that this share increased significantly from 46.9% in the pre-COVID period (before January 31, 2020, when the first two cases were reported in Italy) to 49.5% in the post-COVID period^14^. For regional news the jump was even more significant, going from 12.3 to 15.3%. In absolute numbers, around 10.5 million people per day watch the national news over the entire period, while 2.6 million watch the regional news, out of a total population of around 60 million. The changes between the pre- and post-COVID periods correspond to an additional 2 million viewers for national news and almost 1 million more viewers for regional news.

**Table 1:** Summary Statistics, national data. Daily data on primetime TV news and Covid-19 cases, before Covid-19 versus during Covid-19

Variable	Overall sample		Pre-covid	Post-covid	Difference	t-test
	Obs	Mean (SD)	Mean (SD)	Mean (SD)	Diff. (SE)	t (prob.)
Share of National News^*^	572	47.73	46.92	49.53	2.60	12.09
		(2.67)	(2.01)	(3.05)	(0.21)	(0.0000)
Share of Regional News^**^	573	13.21	12.26	15.31	3.04	24.61
		(1.96)	(0.82)	(2.13)	(0.12)	(0.0000)
Viewers of National News (1000)	572	10,492	9,856	11,898	2,041	10.98
		(2,263)	(1,602)	(2,817)	(185.8)	(0.0000)
Viewers of Regional News (1000)	573	2,619	2,315	3,293	977	16.72
		(789)	(399.5)	(998.5)	(58.45)	(0.0000)
New positives (1000)	573	0.42		1.38		
		(1.15)		(1.72)		
Total current positives (1000)	573	12.82		41.28		
		(28.35)		(37.63)		
New deaths	572	61.37		197.23		
		(165.49)		(247.8)		
Total patients hospitalized (1000)	573	3.19		10.27		
		(7.84)		(11.20)		
Total in intensive care (1000)	573	0.32		1.04		
		(0.86)		(1.29)		

For TGN, there is one observation less since the audience for May 27, 2019 is missing for one channel (TG7). The table reports the number of observations; the mean and standard deviation (in brackets) for the overall sample; the mean and standard deviation (in brackets) for the sample before and from January 31, 2020; the difference in means and standard deviation (in brackets) between the two subsamples pre-post covid; the t-test for difference in means and the probability that Pr(|T| >|t|) under the null assumption that the difference in means is different from zero

^*^National News is the sum of the three main National News broadcasts 8:00–8:30 PM (TG1 + TG5 + TG7)

^**^Regional News is the share of the Regional News broadcast 7:30–8:00 PM (TG3 Regional)

An important feature of the data we use is that they correspond to information disseminated by the news outlets in the corresponding days, thus influencing the perceptions of Italian citizens about the epidemiological developments 15. At times these data have later been revised, but we use them as they were reported. This time series starts on February 24, 2020, just few days after the first documented case of COVID-19 transmission in Italy, on February 21, 2020.

As the main explanatory variables, we use the number of new positives and the number of current positives on any given day. Both measures capture the current intensity of the infection, albeit with a different timeframe, as the variable current positives is the sum of new positives in the past, minus those who recovered or died. In Table 1, we can see that the average number of new positives is 1.38 thousand per day over the COVID-19 period, and the number of current positives nationwide averages 41.3 thousand per day. We also report the number of deaths, hospitalizations, and patients in intensive care units, which we use in robustness checks as alternative measures of the seriousness of the pandemic. In the case of deaths, numbers are much smaller and noisy, in particular for smaller and less affected regions. Nationwide, there are on average 197 COVID-related deaths per day in the 178 days of the COVID period we consider, while the average number of hospitalized people is 10.3 thousand and the average number of patients in intensive care units is 1 thousand.

**Fig. 1:**
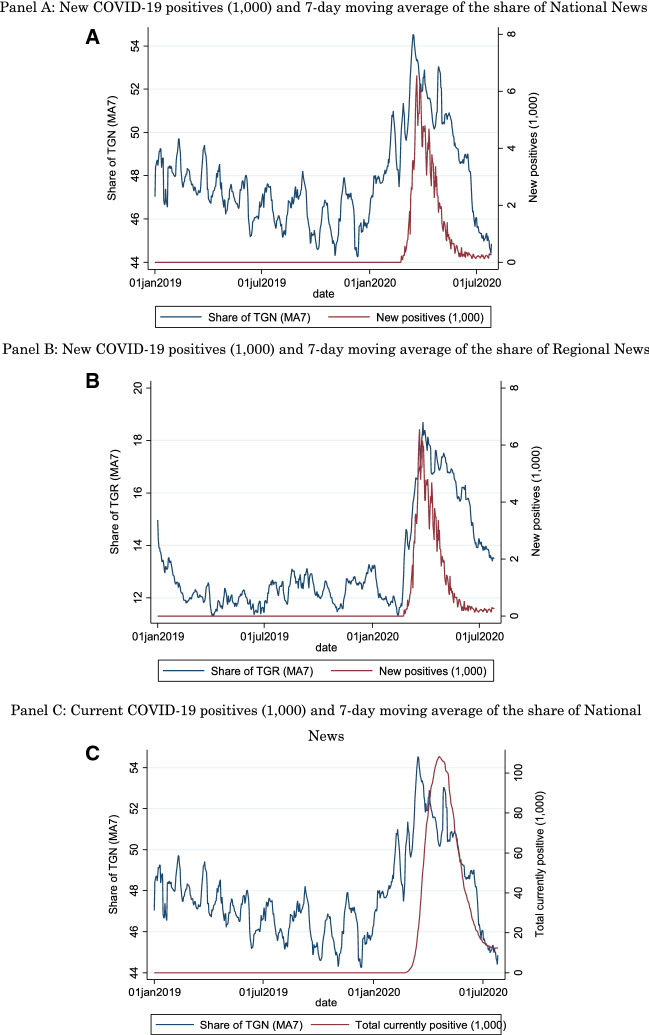

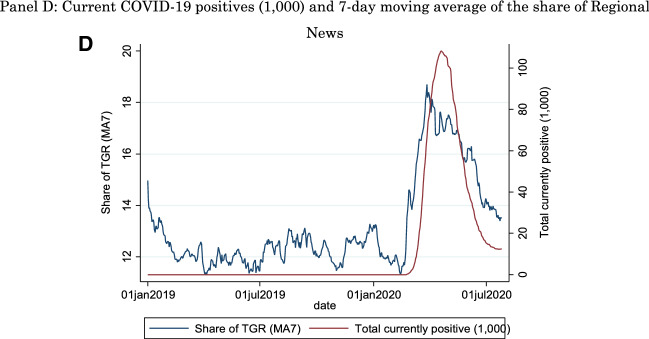
Share of National and Regional News and COVID-19 Developments. Panel A: New COVID-19 positives (1000) and 7-day moving average of the share of National News. Share of TGN is the 7-day moving average (MA7) share of TV viewers watching the National news (TGN). The horizontal axis is time, the left axis is the share of News viewers and the right axis is the number of new positives (in units of 1000). Panel B: New COVID-19 positives (1000) and 7-day moving average of the share of Regional News. Share of TGR is the 7-day moving average (MA7) share of TV viewers watching the Regional news (TGR). The horizontal axis is time, the left axis is the share of News viewers and the right axis is the number of new positives (in units of 1000). Panel C: Current COVID-19 positives (1000) and 7-day moving average of the share of National News. Share of TGN is the 7-day moving average (MA7) share of TV viewers watching the National news (TGN). The horizontal axis is time, the left axis is the share of News viewers and the right axis is the total number of currently positives (in units of 1000). Panel D: Current COVID-19 positives (1000) and 7-day moving average of the share of Regional News. Share of TGR is the 7-day moving average (MA7) share of TV viewers watching the Regional news (TGR). The horizontal axis is time, the left axis is the share of News viewers and the right axis is the total number of currently positives (in units of 1000)

**Fig. 2:**
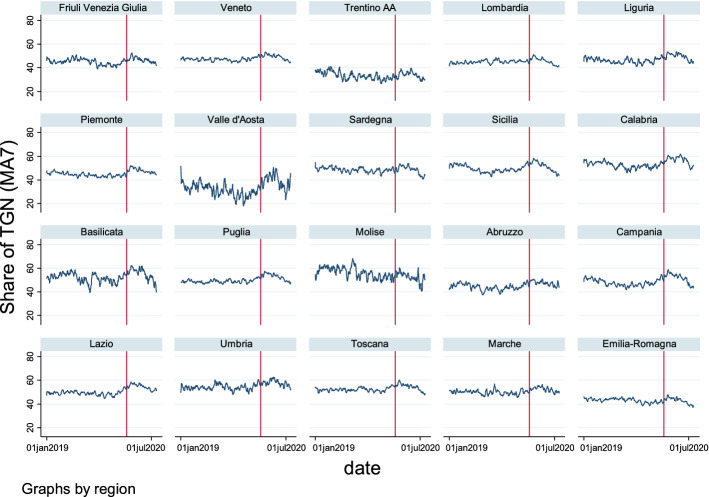
Share of TGN (7-day Moving Average) over time, by region

**Fig. 3:**
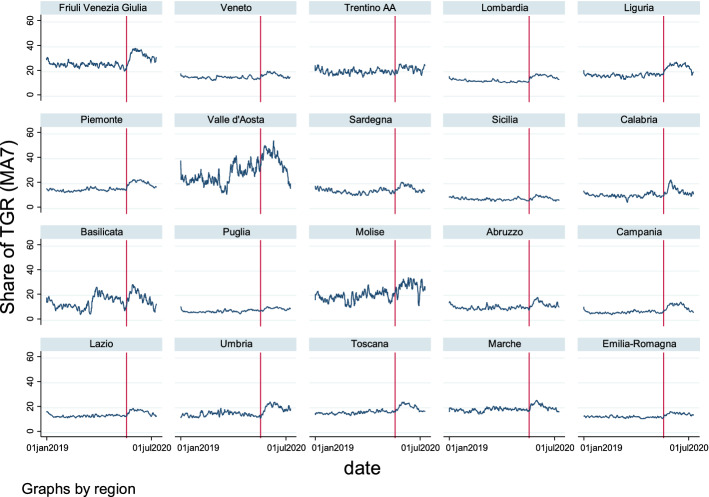
Share of TGR (7-day Moving Average) over time, by region

**Fig. 4:**
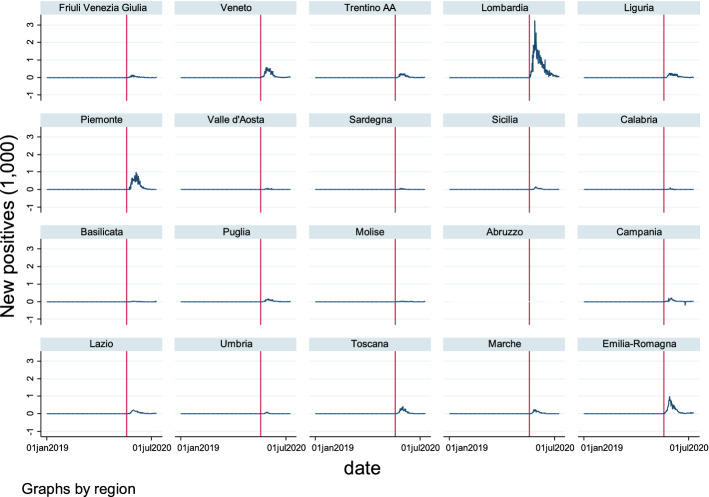
New cases of Covid-19 (1000) over time, by region

**Fig. 5:**
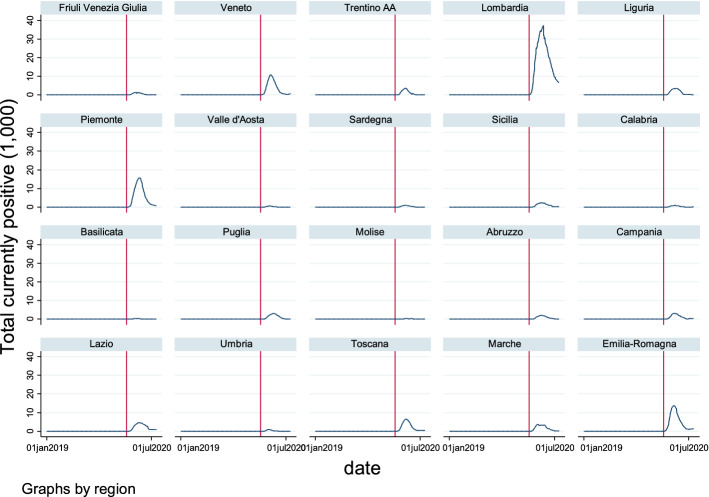
Total cases of Covid-19 (1000) over time, by region

Figure 1, Panel A shows the daily number of new COVID-19 cases in units of one thousand (right axis) and the 7-day moving average of the share of the national news (TGN) (left axis), while the horizontal axis is time. In Panel B, it shows the local primetime news (TGR) in Italy (left axis), while Panels C and D report similar plots with the total number of current positives (in the Appendix, Figs. 2, 3, 4 and 5, we plot the same data at the regional level). It is evident how the period we consider includes both the initial growth of the infection, with a peak in March 2020 for new positives and in April 2020 for current positives, as well as the decline of this first wave. The four plots show an upward trend of the share of national and local news as public health conditions worsened, and a downward trend when, after the imposition of a lockdown, the situation improved. One notes how the increase in the share of regional news closely tracks the increase in positives, while national news already appears to be trending upward before the start of the pandemic in Italy. This would be consistent with a heightened attention to the international aspects of the pandemic (i.e., the developments in the Chinese region of Wuhan), attention that would naturally be satisfied by national news also covering international events, but not by regional news. Moreover, it appears that the decline in share tracks the number of current positives more closely than the number of new positives.

As mentioned, the panel we use covers all 20 Italian regions. The epidemiological developments have differed greatly, with a notable North–South gradient. Lombardy, as noted, has been the hardest hit region by far, but other northern regions like Piemonte, Veneto and Emilia-Romagna have also suffered heavily. In our data, these regions, each with over 4 million inhabitants, have an average of over 100 new positives per day, while in southern regions like Campania, Puglia and Sicilia, with over 4 million inhabitants each, the number is below 30 (see Table 2). There is also some heterogeneity regarding the share of national news. Excluding Valle d’Aosta and Trentino-Alto Adige, where there are consistent linguistic minorities and the share of national news is just above 30%, in the rest of the country the average daily share in the pre-COVID period ranges between 43 and 57%. For regional news (provided in standard Italian as well as minority languages) the range is wider, spanning from 6% in Campania to 26% in Friuli-Venezia Giulia.

**Table 2:** Summary Statistics, regional data. Daily data on primetime TV news and Covid-19 cases, averages before Covid-19 versus during Covid-19

	Share of National News^*^		Share of Regional News^**^		New positives	Total current positives (1000)	New deaths	Total patients hospitalized	Total in intensive care	Population (millions)
Region	Pre-covid	Post-covid	Pre-covid	Post-covid	Post-covid	Post-covid	Post-covid	Post-covid	Post-covid	1 Jan 2020
Valle d'Aosta	31.0	38.6	25.2	37.9	7	0.1	0.8	0.03	0.00	0.1
Piemonte	44.3	47.4	14.6	19.6	178	5.2	23.2	1.29	0.11	4.3
Liguria	45.9	49.4	16.6	23.1	57	1.2	8.8	0.36	0.04	1.5
Lombardy	45.0	45.6	12.2	15.5	539	16.3	94.4	4.38	0.38	10.1
Trentino-A.A	33.3	33.6	19.7	21.9	41	0.9	3.9	0.17	0.02	1.1
Veneto	47.0	49.2	15.1	16.8	111	3.2	11.6	0.51	0.07	4.9
Friuli-V.G	44.9	46.7	25.6	31.8	19	0.4	1.9	0.07	0.01	1.2
Emilia-Romagna	43.1	42.8	11.5	13.7	166	4.4	24.1	1.19	0.11	4.5
Tuscany	52.1	54.5	15.1	19.1	59	1.9	6.4	0.35	0.07	3.7
Marche	49.8	52.2	17.3	19.5	38	1.4	5.5	0.31	0.04	1.5
Umbria	54.0	57.7	13.9	18.5	8	0.2	0.4	0.05	0.01	0.9
Lazio	49.1	54.3	12.5	15.4	48	1.9	4.8	0.64	0.06	5.9
Abruzzo	44.1	47.4	9.5	12.1	19	0.7	2.6	0.14	0.02	1.3
Molise	56.6	52.7	18.5	25.9	3	0.1	0.1	0.01	0.00	0.3
Campania	47.6	51.8	5.8	9.7	28	1.0	2.4	0.25	0.04	5.8
Basilicata	51.7	54.8	14.0	16.9	3	0.1	0.2	0.02	0.00	0.6
Puglia	49.0	52.7	6.7	8.7	26	1.0	3.1	0.21	0.02	4.0
Calabria	52.3	56.4	10.0	13.3	7	0.3	0.5	0.05	0.00	1.9
Sicily	49.1	51.4	7.1	7.7	20	0.8	1.6	0.17	0.02	5.0
Sardinia	48.5	48.4	13.3	15.6	8	0.3	0.8	0.05	0.01	1.6
Average	46.9	49.4	14.2	18.1	69	2.1	9.9	0.51	0.05	3.0

For TGN, there is one observation less since the audience for May 27, 2019 is missing for one channel (TG7). The table reports the mean for the sample before and after January 31, 2020. The average gives equal weight to all regions

^*^National News is the sum of the three main National News broadcasts 8:00–8:30 PM (TG1 + TG5 + TG7)

^**^Regional News is the share of the Regional News broadcast 7:30–8:00 PM (TG3 Regional)

In the following section we analyze the relationship between news viewership and epidemiological conditions within a regression framework.

## Results

Our general specification is the following:

$${y}_{it}={\alpha }_{i}+\sum_{j=1}^{6}{\gamma }_{j}{D\_day}_{j,t}+{\delta }_{1}{D\_holiday}_{t}+{\beta }_{1}{C}_{it}+{\beta }_{2}{\overline{C} }_{it}+{\varepsilon }_{it},$$

where ${y}_{it}$ is the news share (either for national or regional news) observed in region *i* in day *t*. We also include a region fixed effect, ${\alpha }_{i}$, to capture all regional characteristics (e.g., age structure, presence of linguistic minorities) which can be considered invariant in the time period we consider. The regression also includes six dummies for the days of the week, ${D\_day}_{j,t}$, and a national holiday dummy, ${D\_holiday}_{t}$, to capture time variation due, for instance, to availability of alternative TV programs that may be aired on specific days of the week or to differing family schedules during holidays. The coefficients of interests are ${\beta }_{1}$ and ${\beta }_{2}$, capturing the relationship between TV share in the region and COVID-19 developments (e.g., number of new positives) in day *t* in region *i*, ${C}_{it}$, and in other Italian regions, ${\overline{C} }_{it}$, respectively. As a robustness check, we also use a 7-days moving average or a 1-day lag for the epidemiological variables. In all regressions we use standard errors clustered at the regional level, robust to cross-sectional heteroskedasticity and within-panel serial correlation.

In Table 3, we present the coefficients for national news (first three columns) and regional news (last three columns). First, we assess the relationship with new positives and total current positives separately, then in Columns (3) and (6) together in the same regression. In all instances, it appears that what drives TV viewers’ engagement with the news are not the local developments concerning the pandemic, but rather the developments in the country as a whole. This is true for both national and local news. Indeed, the impact of local positives often has a negative sign and is never significant, while the impact of positives in other regions is consistently positive and significant at 1% level^16^. Examining the specifications in Columns (3) and (6), a thousand new positives in other regions imply an increase in the share of national news by 0.9 percentage points and an increase in the share of local news by 1 percentage point. Table 1 indicates that, nationwide, the average number of new positives during the pandemic is 1.38 thousand, with a standard deviation of 1.72. A thousand more total current positives in other regions implies an increase in the share of national news by 0.03 percentage points and of local news by 0.05 percentage points. Nationwide, the average number of total current positives was 41 thousand, with a standard deviation of 38. So, the coefficient for new positives is 30 times (for national news) or 20 times (for local news) higher than the coefficient for total current positives, but the average number of total current positives nationwide is 30 times higher than the average number of new positives. So, the impact of the two variables capturing the epidemiological developments is, broadly speaking, of the same order of magnitude. Also, these magnitudes are in line with the descriptive statistics in Table 1 which showed an increase, between the pre- and post-COVID periods, of 2.6 percentage points in the share of national news and of 3 percentage points in the share of regional news. An important point to keep in mind when assessing these figures is that local news has a much lower base compared to national news, averaging before COVID a 14% share vs 47% for national news.

**Table 3:** Determinants of the share of TV News at primetime

Variables	(1)	(2)	(3)	(4)	(5)	(6)
	National News			Regional News		
New positives in the region (1000)	− 0.421		0.847	− 0.924		− 1.006
	(0.908)		(0.670)	(1.506)		(0.768)
New positives in other regions (1000)	1.308^**^		0.856^**^	1.850^**^		0.979^**^
	(0.177)		(0.102)	(0.265)		(0.190)
Total current positives in the region (1000)		− 0.0579	− 0.0832		− 0.0297	− 0.00123
		(0.0425)	(0.0432)		(0.0715)	(0.0632)
Total current positives in other regions (1000)		0.0514^**^	0.0258^*^		0.0779^**^	0.0488^**^
		(0.00954)	(0.00974)		(0.0119)	(0.0103)
Baseline (average share pre-COVID)	46.9	14.2				
Observations	11,440	11,440	11,440	11,460	11,460	11,460
R2 within	0.10	0.09	0.11	0.19	0.20	0.23
R2 between	0.03	0.02	0.02	0.01	0.02	0.02
R2 overall	0.04	0.04	0.05	0.08	0.08	0.09

For National News, there are twenty observations less since the audience for May 27, 2019 is missing for one channel (TG7). Results come from panel regressions with region fixed effects which also include DVs for day of week and national holiday. Robust standard errors clustered at the regional level in parentheses

^**^p < 0.01

^*^p < 0.05

One might worry that, particularly for the areas hardest hit by the epidemic (i.e., the north) there might be high collinearity between regional and non-regional epidemiological conditions, making it difficult to identify each of their coefficients separately. This is, however, not the case. In the period of the pandemic, the correlation between new positives in the region and in other regions is 0.38 for the northern regions, while the correlation between total current positives in the region and in other regions is 0.25. Another possible concern is that there might be little time variation in local epidemiological conditions within southern regions, where the first wave of the pandemic was rather subdued. The Coefficient of Variation is, however, quite similar between the north and the south, considering both new positives (CV for North: 2,00, for South: 2,04) or total positives (CV for North: 1,63, for South: 1,41).

Given that local news (TGR) follows the TG3 national TV newscast, aired 7:00–7:30 PM on the same channel, RAI3, one might be concerned that our finding is due to inertia: people watch and respond to national epidemiological developments in the national newscast, and then simply continue watching the regional broadcast, whose audience would also depend on national rather than local pandemic developments. To assess whether this is indeed the case, we proceed to calculate the percentage of viewers who watch local news immediately following the national newscast.^17^ This value (for 2020) is, on average, equal to 21.4%, showing that the vast majority of those who watch local news are new viewers, therefore making inertia an unlikely explanation.

Our finding is robust to a series of different specifications reported in Tables 4, 5, 6, 7. First, in Table 4, Columns 1–2, we use the number of per-capita – instead of total – COVID cases, obtained by dividing the number of infections by the population of own (other) region(s). In fact, the same number of COVID cases in more vs. less populated regions could be interpreted in different ways. Adding 1 case in Val D'Aosta (population 0.1 million) is very different than adding 1 case in Lombardy (population 10 million). Having a regional fixed effect in our main specification means that we control for population (and any other regional characteristic that may be considered fixed in the period under review), and the media routinely reported absolute figures about the pandemic (e.g., number of newly infected) and not per capita numbers. Nevertheless, it is reassuring to show that the results are robust even if we consider per capita figures instead of absolute ones.

**Table 4:** Robustness check I

	(1)	(2)	(3)	(4)	(5)	(6)	(7)	(8)
	Per-capita		Month-year DVs		First differences		Moving average 7	
Share of ⇒	National News	Regional News	National News	Regional News	National News	Regional News	National News	Regional News
New positives in the region	3.690	14.64	0.137	− 1.454	0.459	− 0.238	0.911	− 1.235
	(5.654)	(7.506)	(0.461)	(0.847)	(0.445)	(0.567)	(0.754)	(0.940)
New positives in other regions	48.94^**^	39.40^**^	0.146	0.532^**^	0.943^**^	0.324^**^	0.882^**^	1.042^**^
	(7.535)	(10.37)	(0.116)	(0.125)	(0.178)	(0.106)	(0.106)	(0.200)
Total current positives in the region	− 0.273	− 0.00720	− 0.0905^*^	− 0.0113	0.0901	− 0.172	− 0.0844	0.00553
	(0.429)	(0.511)	(0.0423)	(0.0573)	(0.138)	(0.110)	(0.0437)	(0.0611)
Total current positives in other regions	1.469^*^	2.804^**^	0.0185	0.0387^**^	− 0.0396	0.0602^*^	0.0248^*^	0.0470^**^
	(0.596)	(0.615)	(0.0102)	(0.00798)	(0.0195)	(0.0242)	(0.00972)	(0.0102)
N	11,440	11,460	11,440	11,460	11,400	11,440	11,460	11,460
R2 within	0.106	0.224	0.181	0.266	0.03	0.01	0.22	0.39
Dependent variable:	Share		Share		First Difference Share		Share MA7	
Explanatory variables in:	Per-capita^*^ 1000		Levels (1000)		First difference (1000)		MA7 (1000)	

Results come from Fixed-Effects panel regressions which include DVs for day of week and national holiday. Robust standard errors in parentheses. In Columns 3–4 we add month-year DVs

^**^p < 0.01

^*^p < 0.05

**Table 5:** Robustness check II

	(1)	(2)	(3)	(4)	(5)	(6)
	Excluding Lombardy		Excluding Lombardy, keeping cases from Lombardy		Geographical distance	
Share of ⇒	National News	Regional News	National News	Regional News	National News	Regional News
New positives in the region	0.360	− 1.969	− 2.002	− 2.598	1.672	0.508
	(1.173)	(2.182)	(1.383)	(2.256)	(1.014)	(0.891)
New positives in other (neighboring, for Col. 5–6) regions	1.155^**^	1.501^**^	0.950^**^	1.033^**^	0.540	0.313
	(0.174)	(0.331)	(0.120)	(0.229)	(0.367)	(0.306)
New positives in non-neighboring regions					0.896^**^	1.081^**^
					(0.126)	(0.227)
Total current positives in the region	− 0.0913	− 0.133	− 0.0450	− 0.198	− 0.0333	− 0.00633
	(0.176)	(0.251)	(0.176)	(0.244)	(0.0597)	(0.0940)
Total current positives in other (neighboring, for Col. 5–6) regions	0.0462^*^	0.0809^**^	0.0243	0.0549^**^	0.00561	0.0536
	(0.0197)	(0.0212)	(0.0130)	(0.0133)	(0.0212)	(0.0328)
Total current positives in non-neighboring regions					0.0286^*^	0.0475^**^
					(0.0112)	(0.0115)
N	10,868	10,887	10,868	10,887	11,440	11,460
R2 within	0.10	0.21	0.11	0.23	0.11	0.23
Dependent variable:	Share		Share		Share	
Explanatory variables in:	Levels (1000)		Levels (1000)		Levels (1000)	

Results come from Fixed-Effects panel regressions which include DVs for day of week and national holiday. Robust standard errors in parentheses

^**^p < 0.01

^*^p < 0.05

**Table 6:** Robustness check III

Variables	(1)	(2)	(3)	(4)	(5)	(6)
	National News	Regional News	National News	Regional News	National News	Regional News
Total patients in hospitals in the region (1000)	− 0.0547	− 0.0817				
	(0.113)	(0.202)				
Total patients in hospitals in other regions (1000)	0.193^**^	0.285^**^				
	(0.0290)	(0.0404)				
Total in intensive care in the region (1000)			− 0.794	− 1.888		
			(1.340)	(2.448)		
Total in intensive care in other regions (1000)			1.734^**^	2.544^**^		
			(0.256)	(0.373)		
New deaths in the region					0.0346	0.927
					(3.31)	(4.85)
New deaths in other regions					0.00861^**^	0.0127^**^
					(0.00115)	(0.00171)
N	11,440	11,460	11,440	11,460	11,440	11,460
R2 within	0.10	0.21	0.10	0.20	0.09	0.19
Dependent variable:	Share		Share		Share	
Explanatory variables in:	Levels (1000)		Levels (1000)		Levels	

Results come from Fixed-Effects panel regressions which include DVs for day of week and national holiday. Robust standard errors in parentheses

^**^p < 0.01

^*^p < 0.05

**Table 7:** Robustness check IV

	(1)	(2)	(3)	(4)	(5)	(6)
	Moving average explanatory		Lagged explanatory		Data from Feb 25, 2020	
Share of ⇒	National News	Regional News	National News	Regional News	National News	Regional News
New positives in the region	0.746	− 1.295	0.853	− 0.859	0.987	− 1.469
	(0.796)	(1.009)	(0.648)	(0.757)	(0.696)	(0.932)
New positives in other regions	0.897^**^	1.161^**^	0.872^**^	1.009^**^	0.838^**^	0.909^**^
	(0.105)	(0.204)	(0.0979)	(0.188)	(0.113)	(0.195)
Total current positives in the region	− 0.0828	0.0118	− 0.0846	− 0.00380	− 0.0907^*^	− 0.0433
	(0.0438)	(0.0577)	(0.0430)	(0.0640)	(0.0383)	(0.0446)
Total current positives in other regions	0.0227^*^	0.0410^**^	0.0248^*^	0.0471^**^	0.0268^**^	0.0297^**^
	(0.00963)	(0.0101)	(0.00973)	(0.0102)	(0.00601)	(0.00616)
N	11,440	11,460	11,420	11,440	3,080	3,080
R2 within	0.101	0.222	0.106	0.224	0.170	0.204
Dependent variable:	Share		Share		Share	
Explanatory variables in:	MA7 (1000)		Lagged 1 (1000)		Levels (1000)	

Results come from Fixed-Effects panel regressions which include DVs for day of week and national holiday. Robust standard errors in parentheses

^**^p < 0.01

^*^p < 0.05

Next, in Columns 3–4, we repeat regressions 3 and 6 of Table 3, adding Year-Month dummy variables (i.e., dummies for February 2019, March 2019…, January 2020, February 2020…). These variables capture possible seasonal effects on TV viewership, as well as different stages of the pandemic. Even with this demanding specification in which we add time dummies to the regional fixed effects, the results are robust, in particular with reference to regional news.

In Columns 5–6 we estimate a model in which we regress the first difference – defined as the difference between day t and day t– 1 – in the share of national or regional news on a regional fixed effect (thus allowing for a region-specific trend), the day and holiday dummies and the first difference in new positives and current positives. Note that the change in current positives is the sum of the inflow due to new positives and the outflows due to deaths and recoveries. Again, there is no significant impact of the local epidemiological developments, while what happens in the other regions affects how TV viewers’ interest in the news develops, in particular regarding regional news. In Columns 7–8, we smooth out both the share of TV viewers watching the news and the number of people affected by COVID by taking a 7-day moving average. Again, results are robust to this specification.

In Table 5, Columns 1–2, we exclude Lombardy, the most affected region, which accounts for half of overall deaths and 40% of positives. We also exclude cases occurring in Lombardy in the variables “new positives in other regions” and “total current positives in other regions” for the remaining 19 regions, thus considering Italy as if Lombardy were not part of the country. Qualitatively the results remain the same, with larger coefficients for the variables capturing epidemiological developments in other regions. In Columns 3–4 we exclude Lombardy but keep cases occurring in Lombardy within the variables “new positives in other regions” and “total current positives in other regions” for the remaining 19 regions. Results are qualitatively similar. Thus, our finding is not due to the impact in the most affected region.

In the next robustness check, reported in Columns 5 and 6, we delve deeper into the geographical dimension by distinguishing between neighboring and non-neighboring regions. The idea is that people may be particularly sensitive to events in nearby regions, as the virus could easily spread from there, while they may pay less attention to developments occurring further away. We define two regions as neighboring if they share a common border. Some regions like Emilia-Romagna or Lazio share common borders with six other regions, while Sardinia shares none. Sicily is also an island, but being only 3 km away from Calabria, we consider the two regions as neighboring. What emerges is that the share of TV viewers watching the news responds in a significant way to the epidemiological developments in non-neighboring rather than local regions, in which local means within the region or the neighboring regions. This result confirms the lack of importance of proximity for the impact of the pandemic on interest in national and local news.

In Table 6, we use as measures of the epidemiological situation the number of hospitalizations due to COVID (Columns 1–2), the number of patients in intensive care units due to COVID (Columns 3–4), and the number of deaths due to COVID (Columns 5–6), as officially reported on a daily basis during the period under consideration. Once again, for both types of news, interest is not triggered by local events, but rather by what is happening nationally.

This result is also robust to a further series of robustness checks which we perform in Table 7. Considering that there may be a lag in how epidemiological developments affect demand for news, we keep the contemporaneous value for the dependent variable, but use a 7-day moving average (Columns 1 and 2) or a 1-day lag (Columns 3 and 4) for the epidemiological variables. Finally, in Columns 5 and 6, we use only the time period after which the National Department of Civil Protection started reporting cases, beginning on February 24, 2020. In Table 10 of the Appendix, we report a further set of robustness checks in which we include a lagged term of the dependent variable, with a lag of 1 day, 7 days, or 28 days. The results are confirmed. In the fourth section, we discuss the possible reasons for this result, as well as its implications.

Finally, we provide additional evidence using the total number of viewers divided by the population of the region (see Table 11). This is the number of news viewers per capita. This normalization is needed given that the 20 Italian regions have huge differences in terms of population, going from 0.1 to 10 million (see Table 2). It is indeed the case that the number of TV viewers could increase either because of the lookdown forcing people to stay at home, inducing them to watch more TV just because of a lack of alternatives, or because of an increased interest in the news. The data do not allow to distinguish between these two motives.

The reason we preferred looking at the share rather than at the absolute number can be explained by the following example. Suppose that during COVID people watched more TV exclusively because of the first motive (e.g., closure of bars, limitation of interpersonal contacts) and not because of increased interest in the news. Suppose also that, when watching, they spread across the different channels in a way that is similar to the one prevailing in the period before the pandemic, so that some of them end up watching the news, while some end up watching other programmes. As mentioned, this is a situation that cannot be characterized as showing “an increased interest in the news”.

If, under such circumstances, we looked at the absolute number of news viewers during COVID, we would observe an actual increase and it would be a misrepresentation to characterize this increase as “an increased interest in the news”. When looking instead at the share of news viewers, we would show that it stayed constant, correctly reflecting the fact that there was no “increased interest in the news”. This notwithstanding, it can indeed be interesting to explore the effect of COVID cases on the total number of viewers divided by the population of the region. Table 11 confirms previous results.

## Conclusions

While it seems natural that national events draw increased attention towards national news broadcasts, our finding that the case is the same for local news is more surprising. One could argue that epidemics spread and, therefore, people could rightly consider epidemiological developments outside of their own region as highly relevant for what will eventually happen (and may find it relatively easier to get information about national developments from sources other than TV, e.g., internet). As a response, they may follow local news more intensively to monitor and verify developments. This may explain why the local news share responds to national events, but it still remains surprising that the local news share does not respond to local events.

One could argue that, despite being the main source of news for local facts in Italy, the local newscast we study may not be considered a reliable source of local news by viewers. We note, however, that both national and local newscasts used the very same official source (the National Department of Civil Protection) for the crucial news in the period we consider, i.e., the epidemiological developments at the national, or, for TGR, at the regional level. More importantly, we documented how the share of local news viewership increased significantly, indicating that people were relying on this source to get information during the pandemic.

To further establish the robustness of this finding, it would be interesting to verify whether this phenomenon is observable in other institutional contexts, i.e., in other countries with a territorially uneven severity of the contagion during the first wave of COVID.

This finding has implications, for instance, regarding incentives for local politicians to put in place preventive measures. If people were not paying attention to local news due to a lack of localized spread of the virus, then they would not be informed of – and therefore also less likely to reward – efforts by local policy makers to counteract the spread of the pandemic.^18^ Moreover, the fact that viewers devote increased attention to local news in response to national developments may indicate that people are likely to take the national epidemiological developments as counterfactual. In other words, if people in other regions see that in Lombardy the virus is widespread, they may think that, without major policy interventions, the situation would have been the same in their local area. This consideration would deter possible accusations of overreaction in the case of strong local policy measures successfully controlling the pandemic. These two mechanisms suggest that local politicians would be rewarded for their efforts, even if the local epidemiological situation was not threatening^19^, thus escaping the so-called “preparedness paradox” (Kayyem, 2022), a situation that emerges when preventative measures are successful in avoiding damage but are then perceived as unnecessary because the damage never manifested itself. Following the news may indeed help people understand that strong policy reactions were instrumental in limiting the worst consequences of the pandemic in terms of health. 

## Data Availability

In terms of data availability, the data on TV viewership are owned by Auditel (www.auditel.it) and interested researchers should contact the company to obtain them. The remaining data are available from the corresponding author on reasonable request.

## Appendix 1

### Detailed chronology of COVID-19 events and policy responses in Italy

The Italian government declared a six-month state of emergency in response to the COVID-19 outbreak on January 31, 2020, after blocking air traffic from China the day before. Cases of contagion in the northern regions of the country rose more rapidly than in the rest of the country, which led to a series of national and local government measures being implemented concurrently. In the most affected region, Lombardy, the government suspended most public activities, including economic and educational services, in ten villages in Lombardy, with similar measures being adopted in one village in the Veneto region the following day. On February 23, restrictions in these villages were tightened further, including a prohibition to enter or leave the area or hold any type of meeting for the following fourteen days.

On the same day, several regions in the north of Italy suspended upcoming public events and closed schools and museums until Sunday, March 1 for Lombardy, Veneto, Friuli-Venezia Giulia, and Emilia Romagna, and until February 29 for Piemonte, with the provision that the deadline might change according to the development of “epidemiological scenarios”.

On February 24, other northern regions adopted similar isolation measures, such as Liguria and the Province of Trento, followed by the central region of Marche (announcing a preliminary end date of March 4). On March 1, the Government issued a decree suspending public events and closing schools until March 8 in Lombardy, Veneto and Emilia Romagna and in some provinces of Marche and Liguria. On March 4, school closures were extended throughout the whole country until March 15.

On March 8, the government implemented a total lockdown and banned all individual movements with an exception for work or health reasons or for necessity (e.g., purchasing of food and medicines) throughout Lombardy and in certain districts in Emilia Romagna, Veneto, Marche and Piemonte, for a total of 14 districts in the north of the country. The following day the government extended these measures to the whole country. These restrictions were announced to be in effect until April 3. On March 11th, the government also ordered the closure of most retail shops until March 25, with the exception of grocery stores and pharmacies. This included restaurants, bars, and most personal services (such as hair salons).

On March 22, the government announced that the originally scheduled end date for the closure of commercial activities (March 11) was extended to April 3, and it further suspended commercial and industrial activities, prohibiting individual movements outside the town of residence, with exceptions for work or health reasons or for absolute necessity.

On April 1, the government extended a total lockdown to the whole country until April 13, and on April 10 it was prolonged until May 3.

On April 26, the government announced a starter plan for the so-called “Phase 2”, which would begin on May 4. Under “Phase 2”, movement across regions would still be forbidden, while movement between municipalities would be allowed only for work and health reasons, as well as for family visits. Manufacturing industries and construction sites were allowed to reopen.

On May 13, the government announced schools would remain closed until September.

On May 16, the Prime Minister announced the government’s plan for the easing of restrictions. Under this plan, most businesses could reopen, and free movement was granted to all citizens within their region; inter-regional travel was not permitted except in cases of absolute necessity. Swimming pools, gyms, and later theatres and cinemas could also reopen.

On June 3, the government allowed unrestricted travel to and from EU countries and between Italy's regions. The inter-regional and foreign travel ban remained in place until after Italy's June 2 Republic Day holiday, avoiding any mass travel over that long holiday weekend.

### Timeline of COVID-19 epidemic and policy responses in Italy

**Table:**

Date	Event
30-Jan-20	Italy bans flights from China
31-Jan-20	First two cases of COVID-19 diagnosed in Rome
31-Jan-20	Government declares state of emergency
21-Feb-20	First cases of community transmission reported in Lombardy and Veneto; first COVID-19 death (in Vo', Veneto)
21-Feb-20	Most public activities suspended in outbreak areas in Lombardy and (the following day) in Veneto
23-Feb-20	Complete lockdown of outbreak areas in Lombardy and Veneto
24-Feb-20	Schools closed in Lombardy, Veneto, Friuli-Venezia Giulia, Emilia Romagna and (on the following days) Liguria and Marche
4-Mar-20	School closures throughout the whole country, preliminarily ending on March 15
8-Mar-20	Lockdown (“stay at home” measures) declared for Lombardy and 14 districts in Veneto, Emilia Romagna, Piemonte and Marche
9-Mar-20	Lockdown (“stay at home” measures) extended to the whole country until April 3; school closures extended to the whole country until April 3
11-Mar-20	Government ordered closure of most retail stores (exceptions included groceries and pharmacies), restaurants and bars, as well as most personal services until March 25
19-Mar-20	Italy surpasses China as the country with the most reported COVID-19 deaths
22-Mar-20	Government suspended all non-essential economic activities until April 3. It also prohibited individual movements outside people's town of residence (with exceptions for work- and health-related reasons or in the case of absolute urgency). All measures are in effect until April 3
1-Apr-20	Lockdown extended throughout the whole country until April 13
10-Apr-20	Lockdown extended throughout the whole country until May 3
26-Apr-20	Government announced a starter plan for the so-called "Phase 2", beginning on May 4
4- May-20	“Phase 2” began: movements between regions were forbidden, while movements between municipalities were allowed only for work and health reasons, as well as for family visits. Manufacturing industries and construction sites reopened
13-May-20	Government announced schools would remain closed until September
16-May-20	Prime Minister announced the government’s plan for easing restrictions. Under the plan, most businesses could reopen, and free movement was granted to all citizens within their region; movement between regions was still banned for non-essential activities
3-Jun-20	Government allows travel to and from Italy and between the country's regions

See Figs. 2, 3, 4 and 5.

See Tables 8, 9, 10 and 11.

**Table 8:** Summary Statistics, seven National News outlets. Daily data on thousand viewers ("1000 Viewers") and share ("Mean share") of primetime TV news, *before* Covid-19 versus *during* Covid-19

Variable	Overall sample			Pre-covid	Post-covid	Difference	t-test
	Obs	1000 Viewers (SD)	Mean share (SD)	Mean share (SD)	Mean share (SD)	Diff. Mean Share (SE)	t (prob.)
TG1	573	5,083	23.28	22.74	24.46	1.71	12.30
		(1,128)	(1.74)	(1.43)	(1.77)	(0.13)	(0.0000)
TG2	573	1,468	6.89	6.68	7.36	0.67	6.43
		(452)	(1.20)	(1.10)	(1.29)	(0.10)	(0.0000)
TG3	573	1,963	11.35	10.82	12.53	1.71	17.83
		(564)	(1.32)	(0.94)	(1.28)	(0.09)	(0.0000)
TG4	573	630	3.72	3.70	3.77	0.07	2.12
		(163)	(0.39)	(0.38)	(0.41)	(0.03)	(0.0343)
TG5	573	4,210	19.01	18.65	19.80	1.14	11.28
		(933)	(1.24)	(0.98)	(1.39)	(0.10)	(0.0000)
TG6	570	824	5.79	5.65	6.09	0.44	5.86
		(240)	(0.86)	(0.84)	(0.81)	(0.07)	(0.0000)
TG7	572	1,198	5.43	5.51	5.26	− 0.25	− 3.30
		(291)	(0.85)	(0.88)	(0.75)	(0.07)	(0.0010)

For TG6 there are three while for TG7 there is one observation less, because the audience on certain days is missing. The table reports the number of observations; the mean and standard deviation (in brackets) for the overall sample; the mean and standard deviation (in brackets) for the sample before and from January 31, 2020; the difference in means and standard deviation (in brackets) between the two subsamples pre-post covid; the t-test for difference in means and the probability that Pr(|T| >|t|) under the null assumption that the difference in means is different from zero

**Table 9:** Determinants of the share of the seven National News outlets

Variables	(1)	(2)	(3)	(4)	(5)	(6)	(7)
	TG1	TG2	TG3	TG4	TG5	TG6	TG7
New positives in the region	− 0.505	0.344	0.0694	0.444*	0.737	− 0.00388	0.615
	(0.758)	(0.262)	(0.410)	(0.180)	(0.423)	(0.220)	(0.381)
New positives in other regions	0.508^**^	0.266^**^	0.433^**^	0.0152	0.328^**^	0.153^*^	0.0200
	(0.130)	(0.0801)	(0.123)	(0.0488)	(0.0954)	(0.0600)	(0.0544)
Total current positives in the region	− 0.00146	0.0578^*^	− 0.00443	− 0.00943	− 0.0231	0.0648^**^	− 0.0586^**^
	(0.0271)	(0.0205)	(0.0639)	(0.0116)	(0.0346)	(0.0226)	(0.0186)
Total current positives in other regions	0.0101	0.000340	0.0297^*^	0.00318	0.00854	− 0.00413	0.00711
	(0.00599)	(0.00424)	(0.0122)	(0.00303)	(0.00717)	(0.00371)	(0.00491)
N	11,460	11,460	11,460	11,460	11,460	11,400	11,440
R2 within	0.046	0.093	0.121	0.008	0.033	0.011	0.087

Results come from Fixed-Effects panel regressions which include DVs for day of week and national holiday. Robust standard errors in parentheses

^**^*p* < 0.01

^*^*p* < 0.05

**Table 10:** Determinants of the share of primetime TV News, with lagged dependent variable

Variables	(1)	(2)	(3)	(4)	(5)	(6)
	National News			Regional News		
Lag of Y	1 day	7 days	28 days	1 day	7 days	28 days
New positives in the region	0.574	0.506	0.806	− 0.603	− 0.726	− 1.112
	(0.488)	(0.551)	(0.648)	(0.437)	(0.537)	(0.862)
New positives in other regions	0.620^**^	0.637^**^	0.875^**^	0.609^**^	0.765^**^	1.166^**^
	(0.0678)	(0.0714)	(0.0977)	(0.0912)	(0.115)	(0.198)
Total current positives in the region	− 0.0586	− 0.0595	− 0.0830*	− 0.000174	0.00392	0.0128
	(0.0319)	(0.0326)	(0.0370)	(0.0371)	(0.0401)	(0.0438)
Total current positives in other regions	0.0174^*^	0.0172^*^	0.0166	0.0282^**^	0.0277^**^	0.0262^**^
	(0.00749)	(0.00758)	(0.00915)	(0.00621)	(0.00737)	(0.00842)
N	11,400	11,280	10,860	11,440	11,320	10,900
R2 within	0.188	0.180	0.141	0.351	0.313	0.269

Results come from Fixed-Effects panel regressions which include DVs for day of week and national holiday. Robust standard errors in parentheses

^**^*p* < 0.01

^*^*p* < 0.05

**Table 11:** Determinants of the number of TV News viewers per capita

Variables	(1)	(2)	(3)	(4)	(5)	(6)
	Viewers of National News (per capita)			Viewers of Regional News (per capita)		
New positives (1000)	0.0115		0.00147	− 0.00463		− 0.00626
	(0.0124)		(0.0153)	(0.00794)		(0.00630)
New positives other (1000)	0.0215^***^		0.0208^***^	0.0129^***^		0.0110^***^
	(0.00197)		(0.00139)	(0.00140)		(0.00126)
Total current positives (1000)		0.000689^**^	0.000629^*^		− 8.52e-05	8.82e-05
		(0.000297)	(0.000352)		(0.000264)	(0.000198)
Total current positives other (1000)		0.000655^***^	3.32e-05		0.000437^***^	0.000110^***^
		(8.65e-05)	(6.84e-05)		(5.04e-05)	(3.34e-05)
Monday	0.0179^***^	0.0164^***^	0.0178^***^	0.00200^**^	0.00108	0.00184^*^
	(0.00218)	(0.00219)	(0.00222)	(0.000885)	(0.000867)	(0.000879)
Tuesday	0.0115^***^	0.0101^***^	0.0114^***^	0.000140	− 0.000647	5.66e-06
	(0.00217)	(0.00218)	(0.00220)	(0.000785)	(0.000793)	(0.000786)
Wednesday	0.00959^***^	0.00939^***^	0.00958^***^	2.65e-05	− 8.69e-05	1.19e-05
	(0.00179)	(0.00179)	(0.00179)	(0.000796)	(0.000797)	(0.000796)
Thursday	0.00568^**^	0.00637^***^	0.00572^**^	− 0.00169^*^	− 0.00129	− 0.00162
	(0.00210)	(0.00211)	(0.00209)	(0.000957)	(0.000961)	(0.000957)
Friday	0.00199	0.00263	0.00202	− 0.00292^***^	− 0.00255^**^	− 0.00286^**^
	(0.00161)	(0.00163)	(0.00160)	(0.00102)	(0.00102)	(0.00102)
Saturday	− 0.00863^***^	− 0.00758^***^	− 0.00858^***^	− 0.00593^***^	− 0.00534^***^	− 0.00585^***^
	(0.00142)	(0.00148)	(0.00143)	(0.000911)	(0.000891)	(0.000904)
Holiday	− 0.00857^***^	− 0.00973^***^	− 0.00885^***^	0.00289^***^	0.00196^***^	0.00241^***^
	(0.00114)	(0.00117)	(0.00116)	(0.000650)	(0.000620)	(0.000635)
Constant	0.167^***^	0.168^***^	0.167^***^	0.0489^***^	0.0489^***^	0.0484^***^
	(0.00174)	(0.00175)	(0.00179)	(0.000808)	(0.000824)	(0.000846)
Observations	11,440	11,440	11,440	11,460	11,460	11,460
R-squared	0.32	0.20	0.32	0.40	0.28	0.41

Results come from Fixed-Effects panel regressions which include DVs for day of week and national holiday. Robust standard errors in parentheses

^***^*p* < 0.01

^**^*p* < 0.05

^*^*p* < 0.1

## Footnotes

1 The World Health Organization (2020) has recently underscored that “The 2019-nCoV outbreak and response has been accompanied by a massive ‘infodemic’: an over-abundance of information – some accurate and some not – that makes it hard for people to find trustworthy sources and reliable guidance when they need it”.

2 PeopleMeters is a technology for measuring TV viewings inside and outside the home, on TV, tablets, PCs, smartphones and game consoles, by detecting real viewings. A digital tag – or SDK – installed directly on all the players from which broadcasters provide content, measures the viewings of any editorial content played and actually watched by individuals on each device, with no intermediation, second by second. For further details, see: https://www.ipsos.com/en/ipsos-encyclopedia-peoplemeter-aka-audimeter.

3 Larcinese et al. (2011) show that the agenda bias of U.S. newspapers in the coverage of unemployment is a function of the national unemployment rate, but not of the local one.

4 Gollust et al. (2019) underline how TV news shapes health policy and practice through surveillance (reporting of information to the public), interpretation (providing context and meaning), and socialization (shaping norms, values, and beliefs).

5 Given the rapid spread of the virus, national conditions may also matter for local news. We discuss this in more detail in the conclusions. Moreover, in one of our robustness checks, we evaluate whether people may be particularly sensitive to what is happening in nearby regions, as the virus could easily spread from those locations.

6 In the first wave of the pandemic, many measures (e.g., lockdown, closure of economic activities) were adopted nationwide (see Appendix for details).

7 https://www.journalism.org/2020/04/29/2-covid-19-both-a-national-and-local-news-story/ [accessed on November 3, 2020].

8 In a similar vein, Delmastro & Zamariola (2020) find that feelings of anxiety and depression among the Italian population were widespread throughout the country and were not correlated with local epidemiological developments.

9 For an example concerning the role of media on influenza vaccination in Italy, see Brilli et al. (2020). Using daily variation in news reports of alleged adverse effects of the influenza vaccination, they show that media reporting decreases flu vaccination by about 2.5%. In a study of Hungarian counties, Bíró & Szabó-Morvai (2021) show a strong positive effect of mass media coverage of the meningococcal disease on the uptake of meningococcal vaccinations, while they do not find evidence of a positive impact of the disease incidence itself.

10 https://www.journalism.org/2020/04/29/3-majority-of-americans-feel-they-need-breaks-from-covid-19-news-many-say-it-takes-an-emotional-toll/ [accessed on November 3, 2020].

11 https://www.journalism.org/2020/10/29/coronavirus-driven-downturn-hits-newspapers-hard-as-tv-news-thrives/ and https://www.wsj.com/articles/local-tv-sees-spike-in-viewers-drop-in-ads-in-coronavirus-crisis-11585915203 [accessed on November 3, 2020].

12 There are four other national TV news programs (TG2 at 20:30 and TG3 at 19:00 with RAI; TG4 at 18:55 and Studio Aperto at 18:30 – which for convenience we label as TG6 – with Mediaset), which are not broadcasted at primetime and reach a smaller viewership. In Table 8 of the Appendix, we report descriptive statistics for each of the seven national TV news programs. Concerning representativeness, the three national TV newscasts considered here cover a broad spectrum of targets in terms of gender (TG1 and TG5 attract a largely female audience, TG7 a more balanced one), age (TG1 and TG7 are most popular among older people, while TG5 has a greater viewership among millennials), and education level (TG1 and TG5 targeting individuals with lower education levels, while TG7 is very popular among university graduates); see Agcom (2019).

13 Agcom (2018a, 2018b) is based on a survey with a sample of over 14,000 individuals, representative of the Italian population at both national and regional levels. The survey was designed to explore the use of all major media simultaneously in order to avoid the issues arising from silos measurement and to calculate cross-media audiences. It should also be noted that the analysis was structured so that all local media outlets were considered, including online media as long as they were associated with a traditional news outlet.

14 From Table 8 in the Appendix, we see that the remaining four national news outlets account for a much lower share, on average 7% each, for a total of 4.9 million average viewers (gross of possible duplications, due to the different time slots in which they are broadcasted), as opposed to the average of 10.5 million viewers for the three news outlets we use (net of duplications, as they are broadcasted in the same time slot).

15 For COVID-19 statistics, we use the official data released every day at 6 PM by the National Department of Civil Protection (Dipartimento della Protezione Civile)

16 In Table 9 in the Appendix, we report this last specification for all seven national news broadcasts separately, showing that the impact of new positives in other regions is consistently positive and significant for five out of seven channels. The coefficients for new positives in the region are all insignificant, with the exception of TG4, the smallest news broadcast in terms of viewers. The picture for total positives is more mixed.

17 In particular, we analyze all viewers who start watching TG3 in the first 5 min of that program (“initial viewers”) and calculate how many of these subsequently watch TGR.

18 Owen et al. (2012) find a relationship between the number of news sources typically consulted and the likelihood of changing attitudes toward global warming based on current weather conditions.

19 See Fazio et al. (2021) for an analysis of Italian citizens’ opinions concerning the government’s management of the crisis.
